# Tuning Interlayer Exciton Emission with TMD Alloys in van der Waals Heterobilayers of Mo_0.5_W_0.5_Se_2_ and Its Binary Counterparts

**DOI:** 10.3390/nano13202769

**Published:** 2023-10-16

**Authors:** Mohammed Adel Aly, Emmanuel Oghenevo Enakerakpor, Martin Koch, Hilary Masenda

**Affiliations:** 1 Faculty of Physics and Materials Sciences Center, Philipps-Universität Marburg, 35032 Marburg, Germany; 2 Department of Physics, Faculty of Science, Ain Shams University, Cairo 11566, Egypt; 3 School of Physics, University of the Witwatersrand, Johannesburg 2050, South Africa

**Keywords:** van der Waals heterobilayers, alloy TMDs, band offset tuning, interlayer excitons

## Abstract

Semiconductor heterostructures have been the backbone of developments in electronic and optoelectronic devices. One class of structures of interest is the so-called type II band alignment, in which optically excited electrons and holes relax into different material layers. The unique properties observed in two-dimensional transition metal dichalcogenides and the possibility to engineer van der Waals heterostructures make them candidates for future high-tech devices. In these structures, electronic, optical, and magnetic properties can be tuned through the interlayer coupling, thereby opening avenues for developing new functional materials. We report the possibility of explicitly tuning the emission of interlayer exciton energies in the binary–ternary heterobilayer of Mo${}_{0.5}$W${}_{0.5}$Se${}_{2}$ with MoSe${}_{2}$ and WSe${}_{2}$. The respective interlayer energies of 1.516 eV and 1.490 eV were observed from low-temperature photoluminescence measurements for the MoSe${}_{2}$– and WSe${}_{2}$– based heterostructures, respectively. These interlayer emission energies are above those reported for MoSe${}_{2}$/WSe${}_{2}$ (≃1.30–1.45 eV). Consequently, binary–ternary heterostructure systems offer an extended energy range and tailored emission energies not accessible with the binary counterparts. Moreover, even though Mo${}_{0.5}$W${}_{0.5}$Se${}_{2}$ and MoSe${}_{2}$ have almost similar optical gaps, their band offsets are different, resulting in charge transfer between the monolayers following the optical excitation. Thus, confirming TMDs alloys can be used to tune the band-offsets, which adds another design parameter for application-specific optoelectronic devices.

## 1. Introduction

The research work on semiconductor-based heterojunctions started with Esaki in the late 1950s, focusing on the tunnel diode [1]. Further studies were independently carried out by Kroemer and Alferov in the early 1960s, focusing on heterostructures for laser applications [2,3], and then extended to other optoelectronic devices such as light-emitting diodes [4,5,6] and high-electron-mobility transistors [7,8,9]. Recently, two-dimensional (2D) transition metal dichalcogenides (TMDs) have sparked significant scientific research interest owing to their exceptional electronic and optical properties at atomic scales [10,11,12,13,14]. These qualities have made them candidates for prospective practical applications in future technologies [15,16,17,18,19,20]. Moreover, the concept of stacking heterostructures based on 2D monolayers has extended their potential applications resulting from the ability to engineer the band structure [21,22,23,24,25,26,27,28,29]. The heterostructure based on atomically thin two-dimensional layers circumvents the lattice mismatch problems [24] encountered when attempting to epitaxially grow conventional III-V semiconductors [30,31]. This is due to the weak van der Waals forces between the TMDs layers. Of great potential importance for 2D material-based photodiodes could be the type II band alignment, stemming from a staggered gap where the electron and the hole are located in different layers after a charge transfer following an external excitation [32]. This spatial separation gives rise to interlayer excitons (ILX), commonly referred to as charge transfer excitons (CTX), which are bound electron–hole pairs with spatially distinct wavefunctions. These ILX are characterized by long exciton lifetimes and limited light–matter interaction [33,34,35,36]. In addition, ILX have exciton binding energies (>100 meV) and oscillator strengths that are lower than those for direct excitons in monolayers. On the other hand, these values are much larger than semiconductor-based heterostructures such as quantum wells and superlattices [30]. In III-Vs, CTX have binding energies of ∼4 meV [37] and ∼30 meV [38,39] in GaAs- and GaN-based quantum wells, respectively. Interlayer excitons typically have longer lifetimes than their intralayer counterparts because the spatial separation reduces the probability of radiative recombination. This property is crucial for novel designs of excitonic devices based on interlayer excitons. As demonstrated by Shanks et al. [40], it is possible to design an interlayer exciton transistor by controlling the potential energy landscape and the flow of interlayer excitons in heterostructures. Furthermore, the spatial separation of the electron and hole wavefunctions in interlayer excitons also increases the chances of achieving a coherent condensate at elevated temperatures [41]. This opens up opportunities for the investigation of condensate-based optoelectronics and enhances the prospects of generating an exciton current [42].

**Figure 1:**
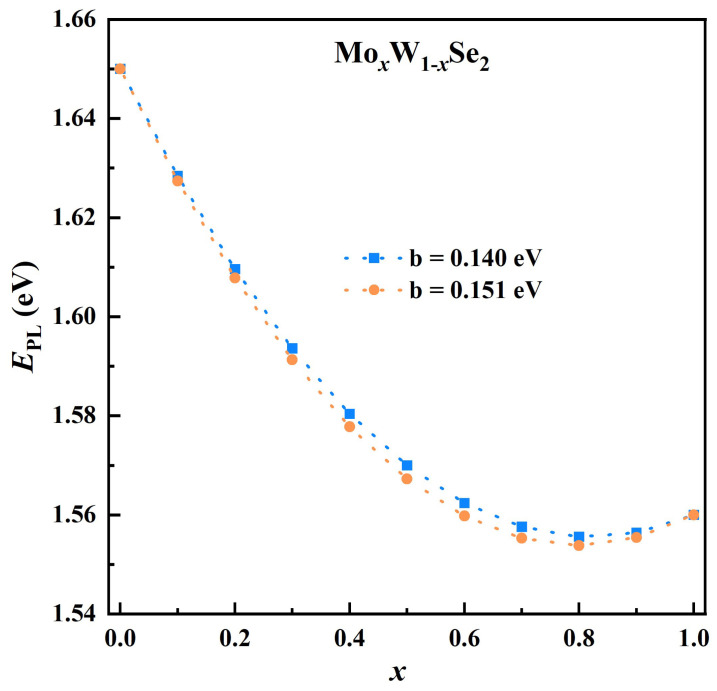
Optical band gap dependence of concentration (*x*) in Mo${}_{x}$W${}_{1-x}$Se${}_{2}$ based on Equation (1) for reported bowing parameters [46,47].

Several computational modelling tools have shown the possible existence of thousands of 2D layered materials [43,44,45]. Cheon et al. [43] reported over 1500 2D materials that can easily be exfoliated with band gaps ranging between 0 and 6 eV. This presents ‘endless’ permutations of potential heterobilayers composed of a pair of different monolayers. An attractive addition to increasing the pool of low-dimensional materials is the use of alloys of existing TMDs. As in III–V semiconductors, alloying offers the modification of lattice parameters and in turn altered material properties, especially of the band gap. Thus, employing alloys of TMDs in van der Waals heterostructures adds another parameter in designing optoelectronics for specific properties. In TMDs, alloying can be achieved via the transition metal or the chalcogen. As demonstrated here and in other studies cited in this report [46,47,48,49,50], the unique monolayer qualities of TMDs such as their direct optical band gaps, high exciton binding energies, and thermodynamic stability are inherited by ternary alloy from their binary TMDs. Tongay et al. [46] demonstrated that the optical band gap variation of Mo${}_{x}$W${}_{1-x}$Se${}_{2}$ does not show a linear dependence with concentrations (*x*). Their result shows that small amounts of Mo have a significant influence on the optical band gap, see Figure 1 The two curves were determined using the equation,

(1) $$E_{\mathrm{PL}}=xE_{\mathrm{PL}}^{{\mathrm{MoSe}}_{2}}+\left(1-x\right)E_{\mathrm{PL}}^{{\mathrm{WSe}}_{2}}-bx\left(1-x\right)$$

where *b* is the bowing parameter with values of 0.14 [46] and 0.151 [47]. The observed bandgap bowing stems from the localization of the conduction band minimum states around *d* orbitals of Mo, while the valance band maximum states are evenly distributed between the *d* orbitals of the two transition metals [46]. In addition, alloying not only allows for a tuning the optical band gap but also for a tuning of the spin-valley effects. Specifically, binary compounds with different transition metals (such as WSe${}_{2}$ and MoSe${}_{2}$) have a spin-orbit coupling of different sizes and signs. Thus, alloying via the transition metal can be used to tune the valley dynamics by controlling the energy difference between the inter- and the intravalley excitons [48]. Consequently, alloy monolayers offer a platform to expand TMD-based heterostructures to binary–ternary and ternary–ternary heterobilayer systems. In this paper, we focus on the binary–ternary systems based on Mo${}_{0.5}$W${}_{0.5}$Se${}_{2}$ and its binary counterparts, namely, WSe${}_{2}$ and MoSe${}_{2}$, i.e., for *x* = 0 and 1.

Recently, Dong et al. [51] could explain the composition dependence of the band edge shift in 2D-TMD alloys by employing the atomic-bond-relaxation (ABR) correlation mechanism within the valance-force-field (VFF) approach. Their finding suggests that the distortion energy stems from the bond-stretching energy when doping with a transition metal, and from the Coulomb electrostatic energy when alloying with the chalcogen. Moreover, this distortion energy of 2D-TMD alloys emanates from the changes in the bond length, the bond angle, and the distribution of atoms, resulting in the shift of bandgap. Since in semiconductor alloys, the distortion energy is equal to the enthalpy of mixing, the bowing parameter is replaced by the interaction parameter (Ω) in their approach. In the case of Mo${}_{x}$W${}_{1-x}$Se${}_{2}$, a value of Ω = 0.167 eV is obtained. This is comparable to the bowing parameters determined experimentally by Tongay 2014 [46] and Zhang 2014 [47].

Over the past decade, Mo- and W-based heterobilayers have been the subject of intense research [33,52,53,54,55,56,57,58,59,60,61,62,63,64,65,66,67,68]. Among these are heterostructures between MoSe${}_{2}$ and WSe${}_{2}$ monolayers [33,56,57,58,59,60,62,64,65,66,67,68]. Band alignments are one of the key factors deciding the desired application for the resulting material structures. The focus has been targeting type II band alignments, which offer interlayer charge transfer. The interlayer exciton emission of the MoSe${}_{2}$/WSe${}_{2}$ heterostructure ranges from ∼1.30 to 1.45 eV, as reported in the articles cited earlier. These are dependent on the stacking order and on the twist angle between the high symmetry points of the respective monolayers. In order to investigate the effect of alloying on the emission energy of the interlayer excitons and other excitonic features, two binary–ternary heterobilayers, MoSe${}_{2}$/Mo${}_{0.5}$W${}_{0.5}$Se${}_{2}$ and WSe${}_{2}$/Mo${}_{0.5}$W${}_{0.5}$Se${}_{2}$, were prepared to target aligning the edges (i.e., approximately aiming for twist angles of 0° or 60°). The optical emission energies for the different excitonic features were studied by photoluminescence spectroscopy.

## 2. Experimental Details

### 2.1. Sample Fabrication

**Figure 2:**
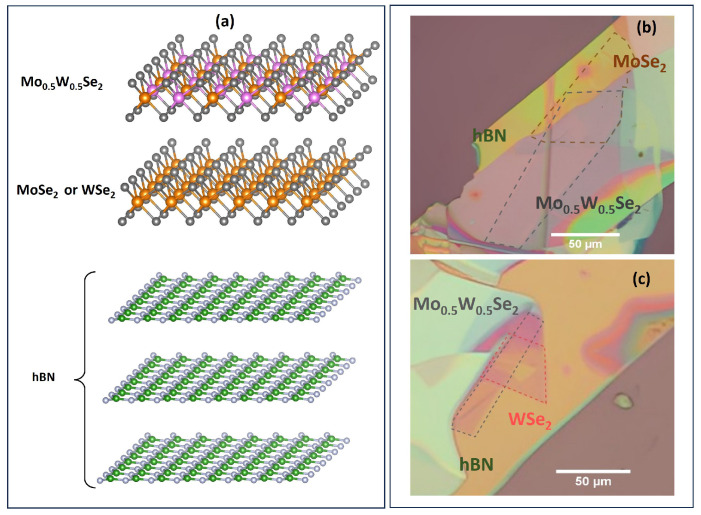
(**a**) Schematic arrangement of heterostructures; (**b**,**c**) optical micrographs of MoSe${}_{2}$/Mo${}_{0.5}$W${}_{0.5}$Se${}_{2}$ and WSe${}_{2}$/Mo${}_{0.5}$W${}_{0.5}$Se${}_{2}$, respectively.

In this investigation, we meticulously prepared the samples by utilizing commercially available bulk crystals of MoSe${}_{2}$ (*HQ Graphene, The Netherlands*) Mo${}_{0.5}$W${}_{0.5}$Se${}_{2}$, and WSe${}_{2}$ (*2D Semiconductors Inc. USA*). The preparation procedure involved the micro-mechanical exfoliation of the bulk crystals. The exfoliated flakes were transferred onto a polydimethylsiloxane (PDMS) gel, which was positioned on a glass slide. The monolayers (MLs) were identified by optical contrast from other flakes of different thicknesses. Prior to the transfer of TMDCs MLs on the target substrate, multiple layers of hBN were transferred on the target substrate (SiO${}_{2}$ (300 nm)/Si) to act as an atomically flat and homogeneous dielectric environment substrate. The selected MLs have been transferred by the viscoelastic stamping technique [69]. After that, the PDMS was slightly lifted and the released MLs remained attached to the target substrate. A schematic representation of the the initially prepared WSe${}_{2}$/Mo${}_{0.5}$W${}_{0.5}$Se${}_{2}$ and MoSe${}_{2}$/Mo${}_{0.5}$W${}_{0.5}$Se${}_{2}$ van der Waals heterostructures (vdW HSs) assembly is depicted in Figure 2a, and the corresponding optical micrographs for the vdW HSs are shown in Figure 2b,c. The interlayer coupling was not significantly strong, likely due to the presence of adsorbates and residues formed at the interface during the transfer process. To address this issue, a thermal annealing process was employed step-wise to eliminate the majority of adsorbates and residuals at elevated temperatures. The as-prepared vdW HSs were initially annealed under vacuum (∼10${}^{-6}$ mbar) conditions at 150 °C for 4 h. However, to achieve further enhancement of interlayer coupling, an additional thermal treatment step was required. Consequently, both heterostructure systems underwent a second annealing process at 300 °C for 4 h.

### 2.2. Optical Characterization

To gain insight into the excitonic properties of the samples, we conducted our measurements using a time-integrated micro-photoluminescence (μ-PL) setup equipped with a nitrogen-cooled charge-coupled device (CCD) Si camera attached to the imaging monochromator (*Princeton Instruments Acton SP2300*). (See Supporting Information for more details on the optical setup). The experiments were performed by employing a continuous wave (CW) laser with an excitation energy of 2.33 eV (532 nm), directed through a 70:30 beam splitter. By finely focusing the laser beam with a power of approximately 360 μW through a 40× objective (NA = 0.6), we were able to obtain a well-defined Gaussian spot with a radius of approximately 2–3 μm. The same microscope objective efficiently collected the PL signal and directed it to the detection path. A 550 nm long-pass filter was placed in the detection path to suppress the laser light in the collected data. Additionally, we incorporated a CMOS camera, along with optical lenses and mirrors, into the experimental setup to facilitate imaging of the monolayers. To maintain precise control over the temperature, the samples were placed within a liquid-Helium-flow microscopy cryostat (*CryoVac*), which allowed us to control the temperature within the range from 10 to 300 K. To correct any aberrations arising from the 1.5 mm thick glass window of the cryostat, we utilized a correction collar associated with the objective.

## 3. Results and Discussion

It is noteworthy that the electronic properties of transition metal dichalcogenides (TMDs), such as MoSe${}_{2}$ and WSe${}_{2}$, are primarily determined by the *d* orbitals of the metal atoms (Mo and W) within their crystal structures. Notably, the energy of the 5*d* orbital of W is higher than that of the 4*d* orbital of Mo, leading to distinct energy band positions in these materials. Specifically, in WSe${}_{2}$, the conduction band (CB) and valence band (VB) are situated at higher energy levels compared to MoSe${}_{2}$ due to the elevated energy of the W 5*d* orbital. However, when Mo${}_{0.5}$W${}_{0.5}$Se${}_{2}$ is considered, it is expected to exhibit intermediate energy band levels between MoSe${}_{2}$ and WSe${}_{2}$, resulting from the mixing of Mo and W orbitals in this alloy. Such unique electronic properties of Mo${}_{0.5}$W${}_{0.5}$Se${}_{2}$ make it a promising candidate for use in heterostructure devices, together with MoSe${}_{2}$ and WSe${}_{2}$.

**Figure 3:**
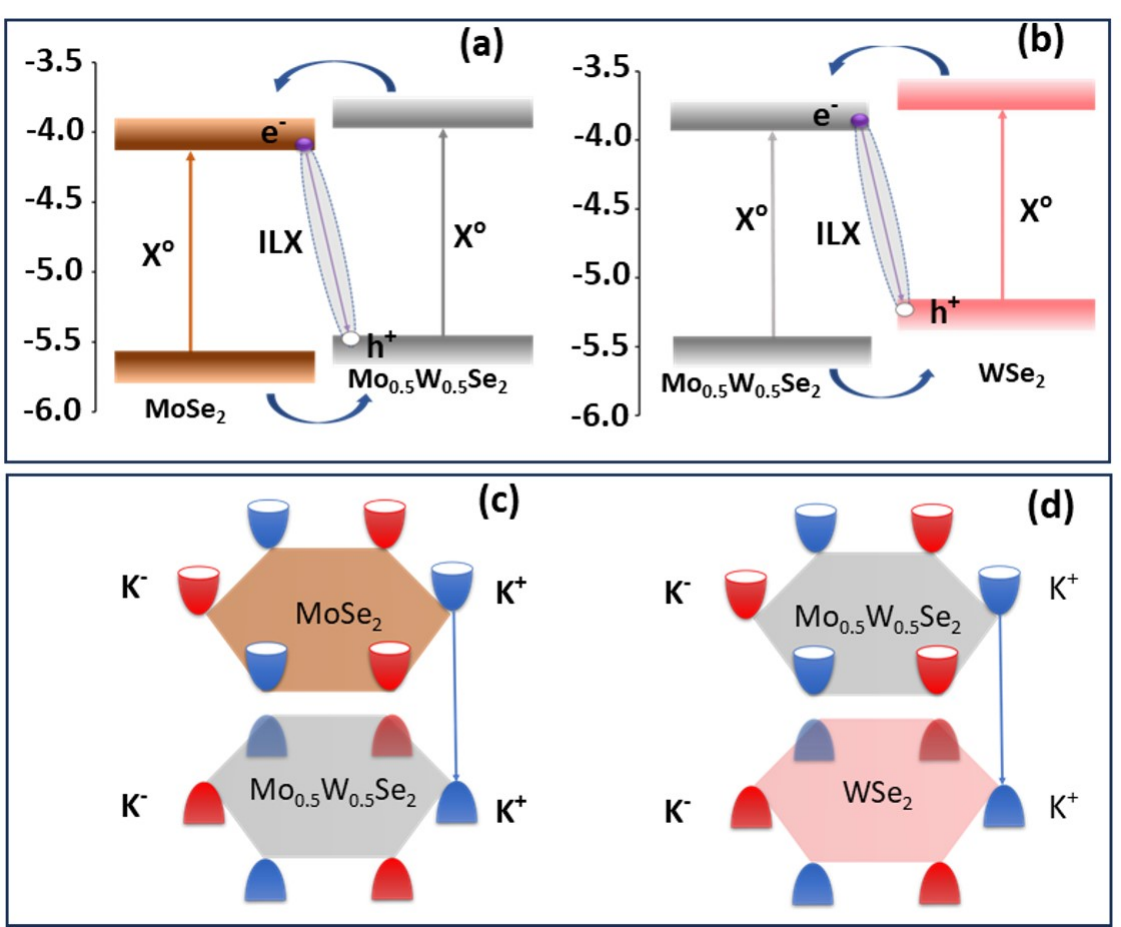
(**a**,**b**) Estimated band alignment in indicated heterostructures (adapted from [49]), with (**c**,**d**) showing schematic representations of the K-K valley transition between corresponding binary–ternary heterostructures.

As highlighted, the heterostructure from binary ends, MoSe${}_{2}$/WSe${}_{2}$, shows a type II band alignment. When fabricating heterostructures based on these TMDs and their alloys, such as MoSe${}_{2}$/Mo${}_{0.5}$W${}_{0.5}$Se${}_{2}$ and WSe${}_{2}$/Mo${}_{0.5}$W${}_{0.5}$Se${}_{2}$, a type II band alignment is expected as depicted in Figure 3a,b respectively. This type of alignment, characterized by staggered band edges, is crucial for the efficient generation and separation of photoexcited carriers in the system. Under photoexcitation, bound electron–hole pairs, known as excitons, are formed in the monolayers of MoSe${}_{2}$, WSe${}_{2}$, and Mo${}_{0.5}$W${}_{0.5}$Se${}_{2}$. The energetic levels of the exciton states are energetically positioned between the VB and CB edges in both layers.

After the optical excitation, the photoexcited electrons and holes undergo relaxation to specific band edges within each material. For instance, in the MoSe${}_{2}$/Mo${}_{0.5}$W${}_{0.5}$Se${}_{2}$ structure, photoexcited electrons relax to the CB edge of MoSe${}_{2}$, while holes relax to the VB edge of Mo${}_{0.5}$W${}_{0.5}$Se${}_{2}$, as shown in Figure 3a. Similarly, in the WSe${}_{2}$/Mo${}_{0.5}$W${}_{0.5}$Se${}_{2}$ structure, electrons relax to the CB edge of Mo${}_{0.5}$W${}_{0.5}$Se${}_{2}$, and holes relax to the VB edge of WSe${}_{2}$, as shown in Figure 3b. This interlayer relaxation process facilitates the spatial separation of photoexcited carriers, which is crucial for the formation of interlayer excitons (colored in gray in Figure 3a,b).

As a final step, when the spatially separated photoexcited carriers recombine across the heterostructure out of these excitonic states, photoluminescence (PL) is emitted as a consequence of the spatially indirect recombination of these interlayer excitons. Due to the type II band alignment, the binding energy of interlayer excitons decreases significantly compared to intralayer excitons, making them less energetically favourable. The binding energy of approximately 100 meV in ILX allows for the efficient emission of photons during recombination, contributing to the observation of photoluminescence in such heterostructures at room temperature.

Importantly, vdW HSs like MoSe${}_{2}$/Mo${}_{0.5}$W${}_{0.5}$Se${}_{2}$ and WSe${}_{2}$/Mo${}_{0.5}$W${}_{0.5}$Se${}_{2}$ exhibit a rapid interlayer relaxation processes, which is approximately 50–100 times faster than the intralayer recombination processes observed in single monolayers [54]. This fast interlayer relaxation ensures the efficient spatial separation of photoexcited carriers, minimizing non-radiative recombination pathways and enhancing the overall optoelectronic performance of the heterostructure [59].

An understanding and the control of interlayer excitons and their dynamics in these alloy-based TMD heterostructures offer potentially promising opportunities for designing novel nanoscale devices with improved efficiency and performance range in various applications, including optoelectronics and quantum information processing [18,70,71,72,73]. Future specific devices will include (but will not be limited to) lasers [74,75], light-emitting diodes [22,58,76], photodetectors in the near- and mid-infrared regions [77,78,79,80,81,82], photovoltaics [83,84,85,86,87], and transistors [35,40,88,89]. These have been demonstrated as proof-of-concepts using binary systems. In addition, because of the inherited spin-valley properties, ILX can be used as spin-valley information carriers, which allows signal processing with reduced power consumption as in current CMOS technology [36]. As highlighted earlier, these spin-valley properties can be tuned in monolayer TMD alloys with different transition metals. Consequently, this, in turn, can influence interlayer valley polarization when incorporated in binary–ternary heterobilayers.

### 3.1. Excitonic Features in Binary–Ternary Heterobilayers

**Figure 4:**
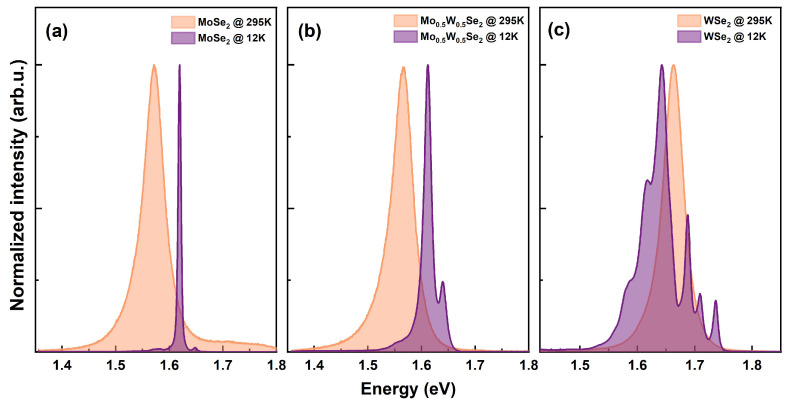
Photoluminescence spectra for the monolayer regions at room temperature and 12K for (**a**) MoSe${}_{2}$, (**b**) Mo${}_{0.5}$W${}_{0.5}$Se${}_{2}$ on MoSe${}_{2}$ HS, and (**c**) WSe${}_{2}$.

Room and low–temperature spectra for monolayers of MoSe${}_{2}$, Mo${}_{0.3}$W${}_{0.7}$Se${}_{2}$, and WSe${}_{2}$ from our fabricated heterostructure systems as described in sample fabrication Section 2.1 are shown in Figure 4. Both systems have undergone annealing at 150 °C for 4 h to enhance the coupling between layers and to release air bubbles at the interfaces. In Figure 4, we present the photoluminescence (PL) signal collected from the three different constituents monolayers for our heterostructure systems, namely (MoSe${}_{2}$, Mo${}_{0.5}$W${}_{0.5}$Se${}_{2}$, and WSe${}_{2}$) at both room temperature and 12 K. The extracted emission energies for excitons (1*s*) and trions are listed in Table 1, for all the monolayer regions including the ternary on both heterostructures. Fitted spectra for the individual monolayers are supplied in the Supporting Information. Figure 4a shows the PL collected from the binary monolayer (MoSe${}_{2}$) region. An obvious blue shift in the excitonic peak position is detected when the temperature changes from room to near liquid helium temperature (12 K) [90,91]. Furthermore, fine structural excitonic features become clearly distinguishable at low temperatures due to the reduction in phonon scattering. At room temperature, only the neutral excitonic peak was detected at 1.572 eV, while the trion peak was hardly resolved. However at 12 K, the neutral exciton was almost completely suppressed, and the trion peak was clearly seen at 1.611 eV. Moreover, the I${}^{T}$/I${}^{o}$ ratio increased from 0.16 to 50 as the temperature changed from room temperature to 12 K. This drastic change in trion contribution can be attributed to the stability of trions at low temperatures and its low binding energy. This was observed before by Duan et al. [92].

Figure 4b presents the excitonic features in the ternary region. At room temperature, the trion peak was significantly suppressed compared to the exciton peak. However, at low temperatures, the trion peak was much more pronounced, and the I${}^{T}$/I${}^{o}$ ratio increased from 0.29 to 5.36. Furthermore, we investigated the WSe${}_{2}$ monolayer, as depicted in Figure 4c. We observed that the I${}^{T}$/I${}^{o}$ ratio increased from 0.12 to 2.6 as the temperature decreased from room temperature to 12 K. From the previous discussions, we can conclude that the intensity ratio for the ternary at 12 K is an average of the values observed for the WSe${}_{2}$ and MoSe${}_{2}$ monolayer regions.

Briefly, MoSe${}_{2}$ and Mo${}_{0.5}$W${}_{0.5}$Se${}_{2}$ are dominated by two prominent features: (i) a most pronounced trion (X${}^{T}$) peak on the low energy side relative to (ii) the neutral exciton (X${}^{o}$). In some cases, a third broad low–energy peak is usually attributed to excitons bound to defects (X${}^{D}$) [93,94]. On the other hand, WSe${}_{2}$ is commonly associated with a number of excitonic features at low temperatures, as reported in recent publications [95,96,97,98,99]. See Supporting Information for fitted spectra and parameters for the additional features. As reflected in Table 1, the peak emission energies for both the ternary monolayers are comparable with values for MoSe${}_{2}$. This agrees with the bowing effect predicted by Tongay et al. [46]. However, even though the optical bandgaps for the 1*s* exciton are similar, their band offsets are different [46,49], a phenomenon attributed to the localization of the CBM state around the Mo *d* orbitals, while the VBM states are uniformly distributed among orbitals of both cations. In the next section, we focus on the effect of different band offsets and interlayer excitons.

### 3.2. Interlayer Exciton-Induced Effects

**Figure 5:**
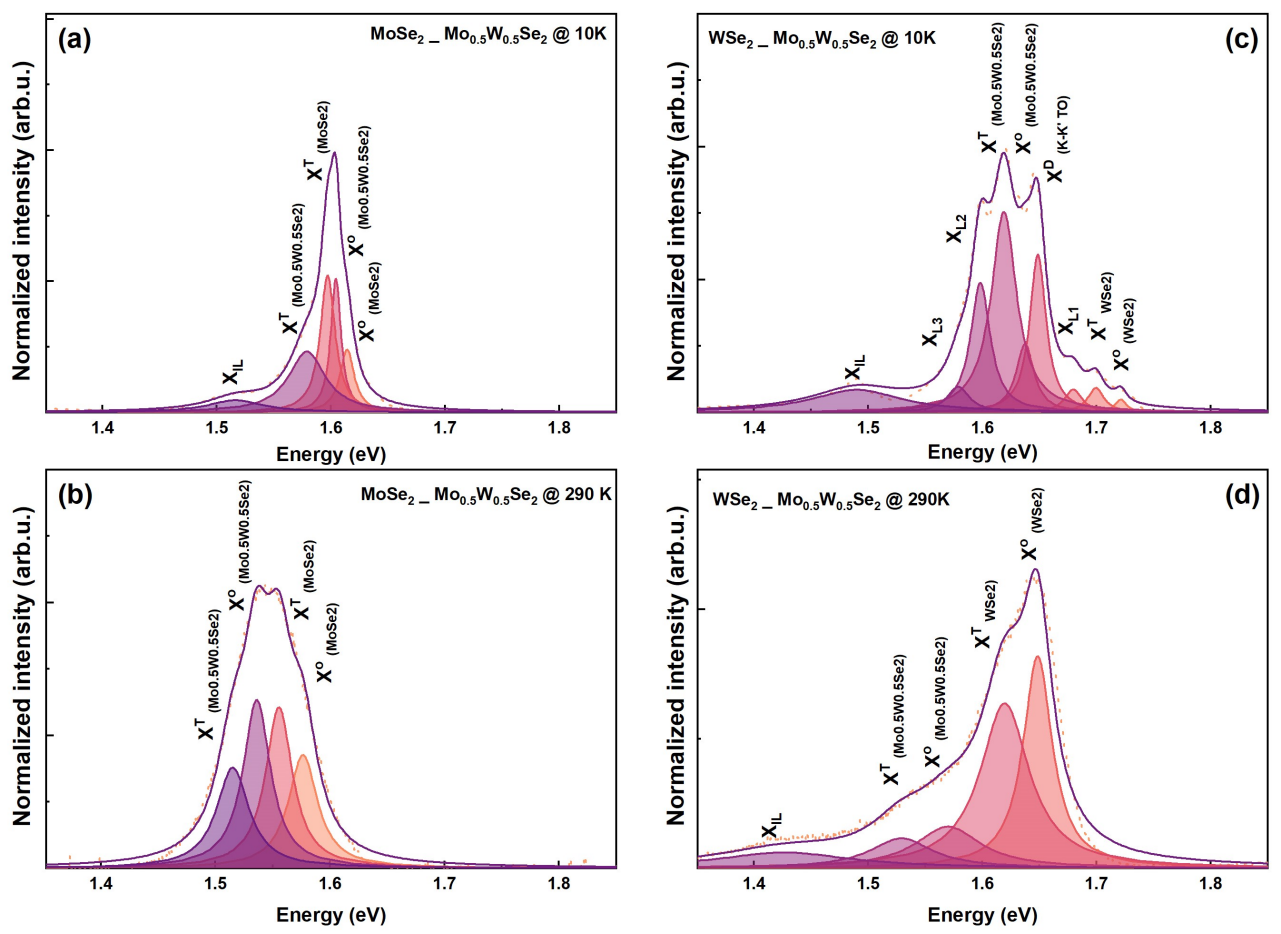
Fitted photoluminescence spectra for the heterobilayers: MoSe${}_{2}$/Mo${}_{0.5}$W${}_{0.5}$Se${}_{2}$ at 10 K (**a**) and 290 K (**b**); and WSe${}_{2}$/Mo${}_{0.5}$W${}_{0.5}$Se${}_{2}$ at 10 K (**c**) and 290 K (**d**).

Initially, we observed a lack of clearly pronounced interlayer exciton signals in samples when annealed at 150 °C, both at room temperature and 12 K. To enhance the coupling between interfaces, we performed a subsequent annealing step at 300 °C for 4 h. We investigate the evolution of photoluminescence (PL) signal from the MoSe${}_{2}$/Mo${}_{0.5}$W${}_{0.5}$Se${}_{2}$ heterostructure spot to study the evolution of excitons at 290 K and 10 K. The data are shown in Figure 5a,b, respectively. Multi-Lorentzian fitting analysis revealed distinct excitonic and trionic features, along with the clear detection of the interlayer exciton (ILX) at 1.51 eV at 10 K. Conversely, at 290 K, we only resolved the neutral and charged exciton peaks for MoSe${}_{2}$ and Mo${}_{0.5}$W${}_{0.5}$Se${}_{2}$, as shown in Figure 5b. The spectra present in Figure 5c depict data collected from the heterostructure spot in WSe${}_{2}$/Mo${}_{0.5}$W${}_{0.5}$Se${}_{2}$ at 10 K, with the successful resolution of multiple excitonic features, including the ILX at 1.49 eV. Additionally, Figure 5d shows the collected spectra for the WSe${}_{2}$-based sample at room temperature, wherein the ILX remains detectable at 1.43 eV, signifying its robustness.

To comprehensively elucidate the origin of interlayer excitons within our heterostructure (HS) system, an exploration of the electronic band structure becomes imperative. Particularly, a precise consideration of the electronic band structure alignment in the WSe${}_{2}$/MoSe${}_{2}$ heterostructure, as outlined by Nayak in 2017 [59], provides invaluable insights. Nayak’s findings emphasize that the evolution of the interlayer peak emanates from K-K transitions across distinct layers, bearing significance in specific electronic bands (see Figure 3c,d). Notably, the valence band maximum (VBM) at the K-point predominantly stems from the WSe${}_{2}$ layer, while the conduction band minimum (CBM) at the K point is largely attributed to the MoSe${}_{2}$ layer. Within such heterobilayer systems, the K point of the bilayer concurs with the K points of individual monolayers, underscoring direct interlayer transitions from the K-point of the valence band to the K-point of the conduction band.

Koo and colleagues [100] have also contributed to our understanding by investigating the interlayer peak evolution in the WSe${}_{2}$/Mo${}_{0.5}$W${}_{0.5}$Se${}_{2}$ configuration. Their analysis reveals analogous mechanics, where the VBM at the K-point is largely governed by the WSe${}_{2}$ layer, while the predominant contribution to CBM at the K-point originates from the Mo${}_{0.5}$W${}_{0.5}$Se${}_{2}$ layer. Furthermore, when considering the MoSe${}_{2}$/Mo${}_{0.5}$W${}_{0.5}$Se${}_{2}$ heterobilayer system, similar mechanisms are anticipated. Here, the VBM at the K-point predominantly originates from the Mo${}_{0.5}$W${}_{0.5}$Se${}_{2}$ layer, whereas the principal contribution to CBM at the K-point originates from the MoSe${}_{2}$ layer.

Further substantiating our comprehension of the K-K transition interlayer exciton is an earlier study by Kunstmann et al. [61]. Their proposition posits the formation of interlayer excitons in the WSe${}_{2}$/MoS${}_{2}$ heterobilayer system as an indirect transition, where the Γ–K transition plays a pivotal role. According to their postulation, a synergistic interplay between charge carriers and a phonon population augments the efficacy of this interlayer exciton (ILX) formation process. Intriguingly, the manifestation of interlayer excitons through the Γ–K transition is anticipated to be notably accentuated at elevated temperatures due to heightened phonon population. Notably, however, this scenario does not align with our heterostructure systems.

The preceding discussion highlights that the interlayer transition in heterobilayer systems originates from the K-K transition within each of the investigated heterostructures (MoSe${}_{2}$/Mo${}_{0.5}$W${}_{0.5}$Se${}_{2}$ and WSe${}_{2}$/Mo${}_{0.5}$W${}_{0.5}$Se${}_{2}$). The observation made in these binary–ternary heterobilayers between Mo${}_{0.5}$W${}_{0.5}$Se${}_{2}$ and its binary counterparts confirms the theoretical predictions reported by Zhou et al. [49]. Concisely, we observed interlayer exciton emission energies at 1.516 eV and 1.490 eV for the MoSe${}_{2}$– and WSe${}_{2}$– based heterostructures with Mo${}_{0.5}$W${}_{0.5}$Se${}_{2}$ as listed in Table 2. These are higher than the ILX energies reported for WSe${}_{2}$/MoSe${}_{2}$ in the range of 1.30–1.45 eV, thus confirming that employing TMD alloys in van der Waals heterostructures can be used to extend the range of emission energies not accessible in binary–binary systems. Furthermore, selecting alloy TMDs with a specific concentration (*x*) adds another design parameter to tune when developing application-desired excitonic devices.

## 4. Conclusions

The concept of type II band alignment in van der Waals heterostructures leading to the formation of interlayer excitons provides opportunities for exploring novel quantum phenomena and developing specialized optoelectronic and quantum information processing devices. Alloyed TMDs inherit direct optical band gaps from their binary counterparts, which are tuned by the relative contributions (*x*) of transition metals. In this paper, we have demonstrated the realization of interlayer excitons in binary–ternary heterobilayers using Mo${}_{0.5}$W${}_{0.5}$Se${}_{2}$ and its binary counterparts, MoSe${}_{2}$ and WSe${}_{2}$. Strikingly, the ternary monolayer, Mo${}_{0.5}$W${}_{0.5}$Se${}_{2}$, and MoSe${}_{2}$ have similar optical band gaps, but different band offsets, which results in type II band alignment resulting in the observation of ILX comparable in energy to the heterobilayer with WSe${}_{2}$. More importantly, the interlayer emission energies observed from the binary–ternary TMD heterobilayers are greater than those in binary–binary systems. Therefore, employing ternary TMDs in heterobilayers not only allows one to tune specific interlayer emission energies but also expands the tuning range of interlayer exciton emission wavelengths. Consequently, alloys of TMDs expand the playing ground and increase the permutation of possible monolayers that can be used for designing application-specific devices. Thus, knowledge of band offsets is vital and adds a critical parameter that can be tuned by alloyed TMDs in heterostructures, thereby modulating interlayer exciton emission in the near-infrared region through band structure engineering.

## 5. Visualization

The schematic depiction of the heterostructure in Figure 2a is based on crystallographic data provided by the Materials Project [101,102,103] and drawn by the VESTA sofware version 3 [104].

## Figures and Tables

**Table 1:** Extracted PL peak energies for observed excitons and trions in monolayer regions.

Monolayer	T [K]	$X^{o}$ [eV]	$X^{T}$ [eV]
MoSe${}_{2}$	300	1.572	1.518
	12	1.640	1.611
Mo${}_{0.5}$W${}_{0.5}$Se${}_{2}$ on ${\mathrm{HS}}_{{\mathrm{MoSe}}_{2}}$	300	1.568	1.545
	12	1.648	1.619
Mo${}_{0.5}$W${}_{0.5}$Se${}_{2}$ on ${\mathrm{HS}}_{{\mathrm{WSe}}_{2}}$	300	1.572	1.538
	12	1.632	1.605
WSe${}_{2}$	300	1.663	1.613
	12	1.736	1.689

**Table 2:** Extracted PL peak energies for observed excitonic features.

MoSe${}_{2}$/Mo${}_{0.5}$W${}_{0.5}$Se${}_{2}$			WSe${}_{2}$/Mo${}_{0.5}$W${}_{0.5}$Se${}_{2}$		
**T [K]**	**10 K**	**RT**	**T [K]**	**10 K**	**RT**
**Peak**	$E_{\mathrm{PL}}$ **[eV]**		**Peak**	$E_{\mathrm{PL}}$ **[eV]**	
$X_{{\mathrm{MoSe}}_{2}}^{o}$	1.614	1.577	$X_{{\mathrm{WSe}}_{2}}^{o}$	1.721	1.649
$X_{{\mathrm{MoSe}}_{2}}^{T}$	1.597	1.555	$X_{{\mathrm{WSe}}_{2}}^{T}$	1.700	1.620
$X_{{\mathrm{Mo}}_{0.5}W_{0.5}{\mathrm{Se}}_{2}}^{o}$	1.604	1.536	$X_{{\mathrm{Mo}}_{0.5}W_{0.5}{\mathrm{Se}}_{2}}^{o}$	1.637	1.570
$X_{{\mathrm{Mo}}_{0.5}W_{0.5}{\mathrm{Se}}_{2}}^{T}$	1.579	1.515	$X_{{\mathrm{Mo}}_{0.5}W_{0.5}{\mathrm{Se}}_{2}}^{T}$	1.619	1.529
-	-	-	$X^{L1}$	1.680	-
-	-	-	$X^{D}$	1.649	-
-	-	-	$X^{L2}$	1.598	-
-	-	-	$X^{L3}$	1.579	-
ILX	1.516	-	ILX	1.490	1.427

## Data Availability

The data supporting this study’s findings are available from the corresponding author upon reasonable request.

## Supplementary Materials

The following supporting information can be downloaded at https://www.mdpi.com/article/10.3390/nano13202769/s1, Figure S1. Schematic diagram of the low-temperature micro-photoluminescence setup; Figure S2. PL spectra of MoSe2/Mo0.5W0.5Se2 HS at Low Temperature; Figure S3. PL spectra of WSe2/Mo0.5W0.5Se2 HS at Low Temperature. Table S1: PL emission energies (in eV) for MoSe2/Mo0.5W0.5Se2-HS at room and low temperatures; Table S2. PL emission energies (in eV) for WSe2/Mo0.5W0.5Se2-HS at room and low temperatures. References [105,106] are cited in the supplementary materials.
