# Supplementary material for: Tuning Interlayer Exciton Emission with TMD Alloys in van der Waals Heterobilayers of Mo0.5W0.5Se2 and Its Binary Counterparts
Source: Nanomaterials (Basel). 2023 Oct 16;13(20):2769. doi: 10.3390/nano13202769 (PMC10609229; doi:10.3390/nano13202769)
Supplement: Supplementary file 1 [file nanomaterials-13-02769-s001.zip › Supplementary Material.pdf]

Supplementary Materials: Tuning interlayer exciton emission with TMD alloys in van der Waals heterobilayers of $Mo_{0.5}W_{0.5}Se_2$ and its binary counterparts

Mohammed Adel Aly [1,2,*], E. O. Enakerakpor [1], Martin Koch [1] and Hilary Masenda [1,3,*]

1. Experimental Set-up

Time-integrated micro-photoluminescence measurements were carried out using the set-up illustrated in Fig S1. An excitation laser light with a wavelength of 532 nm is focused onto the sample using a conventional confocal microscope setup. A 70:30 beam splitter uses a 40× objective (with glass correction) to focus on the sample in a liquid Helium cryostat with a glass window. The PL signal from the sample was collected with the same objective and focused onto the spectrometer slit for the acquisition with a nitrogen-cooled CCD. White light was incorporated in the excitation path for sample imaging using a removable lens and flip mirror coupled with a CMOS camera.

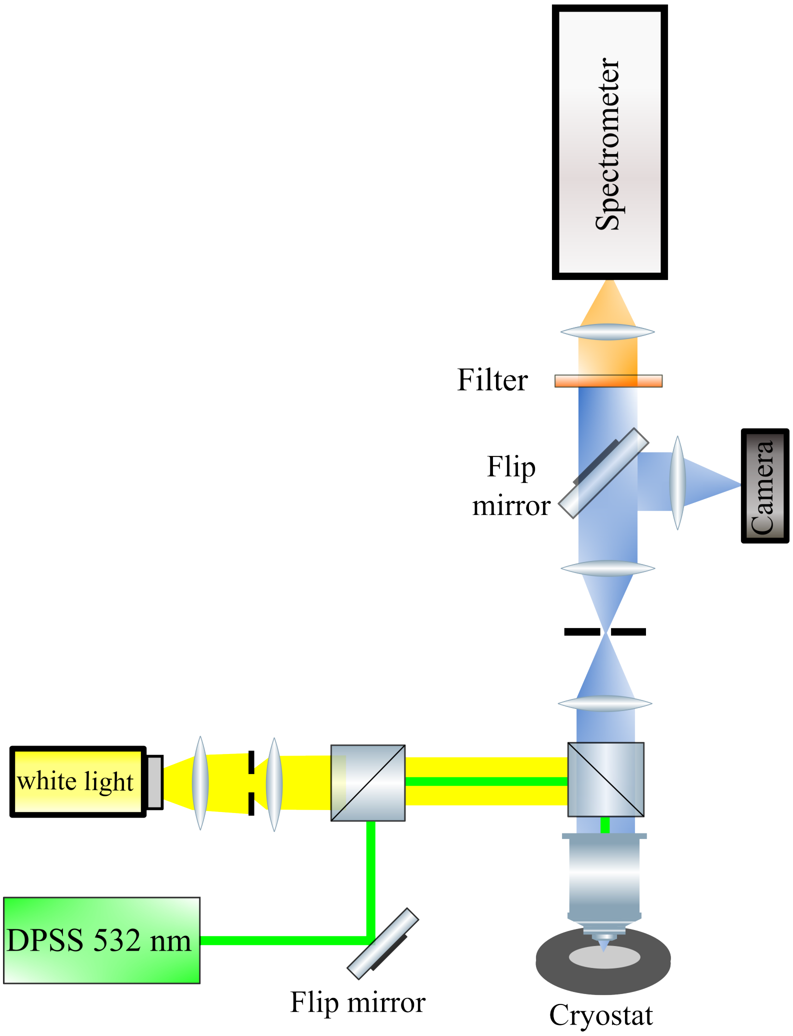

Figure S1. Schematic diagram of the low-temperature micro-photoluminescence setup

2. Additional Spectra

2.1. $MoSe_2/Mo_{0.5}W_{0.5}Se_2$

As highlighted in the main text; the annealing of prepared samples was undertaken stepwise starting with 150°C and then later at 300°C. Fig S2 shows the fitting of the data for the $MoSe_2/Mo_{0.5}W_{0.5}Se_2$ heterostructure after the first annealing step for 4 hours at 150°C. At low temperatures (precisely 12 K), both the binary and ternary parts of $MoSe_2/Mo_{0.5}W_{0.5}Se_2$ have a higher energy peak that is heavily suppressed (especially the $MoSe_2$ counterpart), is the exciton optical bandgap. In addition, there is a very sharp trion peak at a lower energy (Figs S2 (a) and S2 (b). As already mentioned, at cryogenic temperatures a sharp trion peak is often observed because the electrons have less average kinetic energy, leading to reduced collision rates and minimized complexities that could have been caused by thermalization.

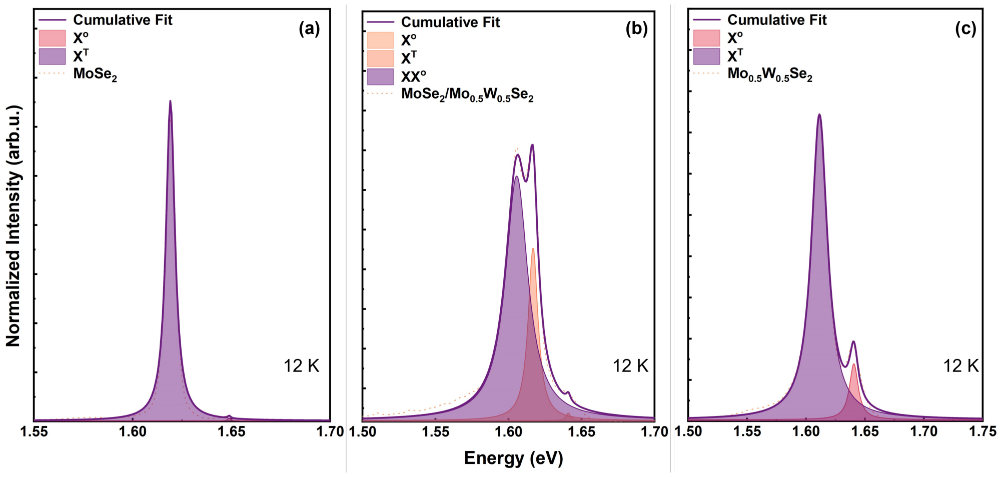

Figure S2. PL spectra of $MoSe_2/Mo_{0.5}W_{0.5}Se_2$ HS at Low Temperature.

These energy peaks (exciton and trion respectively) are present at positions 1.640 eV and 1.611 eV for the binary Fig S2 (a), then 1.648 eV and 1.619 eV for the ternary (Fig S2 (c). The low-temperature and high-temperature PL emission values for the different assigned excitonic features are recorded in Table SI.

Table SI. PL emission energies (in eV) for $MoSe_2/Mo_{0.5}W_{0.5}Se_2$-HS at room and low temperatures.

	**$MoSe_2$**		**$MoSe_2/Mo_{0.5}W_{0.5}Se_2$**		**$Mo_{0.5}W_{0.5}Se_2$**	
**Temperature**	**300 K**	**12 K**	**300 K**	**12 K**	**300 K**	**12 K**
X^o	1.572	1.640	1.538	1.641	1.568	1.648
X^T	1.518	1.611	1.462	1.617	1.545	1.619
X_L				1.606		

2.2. $WSe_2/Mo_{0.5}W_{0.5}Se_2$

At low temperatures, the ternary ($Mo_{0.5}W_{0.5}Se_2$) side of $WSe_2/Mo_{0.5}W_{0.5}Se_2$-HS demonstrated two peaks. These are the exciton and trion peaks at 1.632 eV and 1.605 eV respectively Fig S3 (c). As opposed to this, the binary TMD monolayer (WSe_2) showed more than five peaks as shown in S3 (a). The first two are at 1.736 eV and 1.689 eV; assigned to exciton and trion energy peaks respectively. The remaining peaks at emission energies of 1.709 eV, 1.643 eV and 1.591 eV correspond to the biexciton, dark exciton and local trap states respectively. These energy values alongside others, are given in Table SII.

The numerous energy peaks ranging from 1.564 eV to 1.736 eV, which were demonstrated by the binary counterpart (WSe_2) of the $WSe_2/Mo_{0.5}W_{0.5}Se_2$-HS, having the optical bandgap

of the neutral exciton (X^o) at the highest energy peak of 1.74 eV is similar to values from previous works.

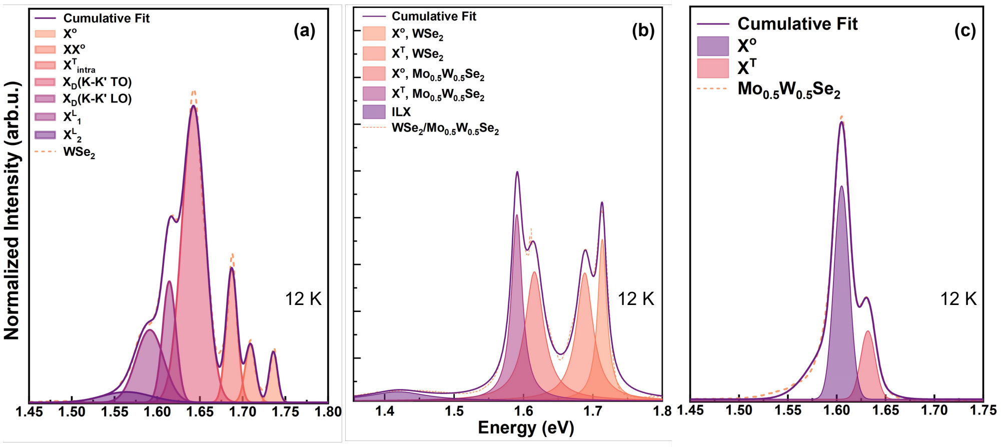

Figure S3. PL spectra of $WSe_2/Mo_{0.5}W_{0.5}Se_2$ HS at Low Temperature.

The spectrum of the HS portion of this sample had five peaks as can be seen in Fig S3 (b). These include four sharp peaks at higher energy values and one not-too-pronounced (suppressed) peak at a lower energy position. The first four higher energy peaks at 1.721 eV, 1.687 eV, 1.616 eV and 1.590 eV correspond to the excitonic and trionic features of the binary (WSe_2) and ternary ($Mo_{0.5}W_{0.5}Se_2$) counterparts respectively. In addition to these four peaks, there is also a lower energy peak at 1.420 eV (as presented in Table SII).

Table SII. PL emission energies (in eV) for $WSe_2/Mo_{0.5}W_{0.5}Se_2$-HS at room and low temperatures.

	**WSe_2**		**$WSe_2/Mo_{0.5}W_{0.5}Se_2$**		**$Mo_{0.5}W_{0.5}Se_2$**	
**Temperature**	**300 K**	**12 K**	**300 K**	**12 K**	**300 K**	**12 K**
X_1^o	1.663	1.736	1.649	1.721	1.572	1.632
X_2^o			1.561	1.616		
XX°		1.709				
X^T	1.613			1.590	1.538	1.605
X_{intra}^T		1.689		1.687		
X^D (K-K′ TO)		1.643				
X^D(K-K′ LO)		1.615				
$X^L{}_1$		1.591				
$X^L{}_2$		1.564				
ILX				1.420		

Interestingly, the entity with the lower energy value at 1.420 eV is that of an interlayer exciton (ILX). It is formed due to a charge transfer between the two spatially separated monolayers (WSe_2 and $Mo_{0.5}W_{0.5}Se_2$), in which the electrons settle at. More precisely, this lower energy peak is believed to be an ILX-2 according to the work of Aubrey *et al.*, [1] who stated that at cryogenic temperature, $WSe_2/MoSe_2$-HS show both ILX-1 and ILX-2.

The heterobilayer part of sample 2 ($WSe_2/Mo_{0.5}W_{0.5}Se_2$-HS) demonstrated both intra and interlayer characteristics and features. Although the suppressed ILE indicates weak interlayer coupling between the constituent monolayers (which could be attributed to strain effects during the fabrication of the vdW-HS) and perhaps other local defects [2–4]. Yet, the presence of the ILE also confirms type II band alignment for $WSe_2/Mo_{0.5}W_{0.5}Se_2$-HS as estimated and predicted previously in this work.

References

1. Hanbicki, A.T.; Chuang, H.J.J.; Rosenberger, M.R.; Hellberg, C.S.; Sivaram, S.V.; McCreary, K.M.; Mazin, I.I.; Jonker, B.T.; Hanbicki, A.T.; Chuang, H.J.J.; et al. Double Indirect Interlayer Exciton in a MoSe2/WSe2 van der Waals Heterostructure. *ACS Nano* **2018**, *12*, 4719–4726. https://doi.org/10.1021/acsnano.8b01369.
2. Tongay, S.; Narang, D.S.; Kang, J.; Fan, W.; Ko, C.; Luce, A.V.; Wang, K.X.; Suh, J.; Patel, K.D.; Pathak, V.M.; et al. Two-dimensional semiconductor alloys: Monolayer Mo1-xW xSe2. *Applied Physics Letters* **2014**, *104*, 12101. https://doi.org/10.1063/1.4834358.
3. Castellanos-Gomez, A.; Buscema, M.; Molenaar, R.; Singh, V.; Janssen, L.; van der Zant, H.S.J.; Steele, G.A. Deterministic transfer of two-dimensional materials by all-dry viscoelastic stamping. *2D Materials* **2014**, *1*, 011002. https://doi.org/10.1088/2053-1583/1/1/011002.
4. Zhu, M.; Zhang, Z.; Zhang, T.; Liu, D.; Zhang, H.; Zhang, Z.; Li, Z.; Cheng, Y.; Huang, W. Exchange between Interlayer and Intralayer Exciton in WSe2/WS2 Heterostructure by Interlayer Coupling Engineering. *Nano Letters* **2022**, *18*, 49. https://doi.org/10.1021/ACS.NANOLETT.2C01353.
